# SeqLengthPlot v2.0: an all-in-one, easy-to-use tool for visualizing and retrieving sequence lengths from FASTA files

**DOI:** 10.1093/bioadv/vbae183

**Published:** 2024-11-20

**Authors:** Dany Domínguez-Pérez, Guillermin Agüero-Chapin, Serena Leone, Maria Vittoria Modica

**Affiliations:** Department of Biology and Evolution of Marine Organisms (BEOM), Stazione Zoologica Anton Dohrn, Calabria Marine Centre (CRIMAC), Località Torre Spaccata, 87071 Amendolara, Italy; PagBiOmicS—Personalised Academic Guidance and Biodiscovery-Integrated OMICs Solutions, Porto 4200-603, Portugal; CIIMAR – Centro Interdisciplinar de Investigação Marinha e Ambiental Universidade do Porto, 4450-208 Matosinhos, Portugal; Department of Biology and Evolution of Marine Organisms (BEOM), Stazione Zoologica Anton Dohrn, Naples 80121, Italy; Department of Biology and Evolution of Marine Organisms (BEOM), Stazione Zoologica Anton Dohrn, Roma I-00198, Italy

## Abstract

**Motivation:**

Accurate sequence length profiling is essential in bioinformatics, particularly in genomics and proteomics. Existing tools like SeqKit and the Trinity toolkit provide basic sequence statistics but often fall short in offering comprehensive analytics and plotting options. For instance, SeqKit is a very complete and fast tool for sequence analysis, delivering useful metrics (e.g. number of sequences, average, minimum, and maximum lengths) and can return sequences either shorter or longer (but not both at once) for a given length. Similarly, Trinity's Perl-based scripts provide detailed contig length distributions (e.g. N50, median, and average lengths) but do not include the total number of sequences or offer graphical representations of the data.

**Results:**

Given that key sequence analysis tasks are often distributed across multiple tools, we introduce **SeqLengthPlot v2.0**, an all-in-one, easy-to-use Python-based tool. Through a simple command-line interface, this straightforward tool enables users to split input FASTA files (nucleotide and protein) into two distinct files based on a customizable sequence length cutoff. It also automatically retrieves the resulting FASTA files, generates length distribution plots, and provides comprehensive statistical summaries.

**Availability and implementation:**

SeqLengthPlot_v2.0.2 can be accessed at https://github.com/danydguezperez/SeqLengthPlot/releases/tag/v2.0.2.

## 1 Introduction

Some tools are available for profiling sequence statistics on FASTA datasets and performing sequence manipulation, such as SeqKit (Shen *et al.* 2016), Seqfu (Telatin *et al.* 2021), or Trinity's utilities (Grabherr *et al.* 2011, Haas *et al.* 2013). These tools are commonly used to profile important statistics on *de novo* transcriptome assemblies.

For instance, SeqKit and Seqfu offer extensive and comprehensive sets of tools. The **seqkit stats** command in the SeqKit package provides information about format (e.g. FASTA or FASTQ), type (DNA, RNA, protein), number of sequences, total bases or residues, and the minimal, maximal, and average sequence lengths for a given input FASTA file. Similarly, Trinity’s **TrinityStats.pl** and Seqfu provide detailed statistics on assembled transcript length distributions, including metrics such as contig length (N10–N50), median length, and average length.

For example, the **seqkit seq** command (**-M** or **-m**) can return sequences shorter or longer than a specific length threshold. However, it cannot split a dataset into both shorter and longer sequences in a single command. While it is possible to combine multiple functions on the same command line (e.g. **cat input_fasta | seqkit seq -m 100 -M 1000 | seqkit stats**) or use existing shell wrapper around seqkit (https://www.biostars.org/p/215287/#319336) and python script (https://github.com/derwiki/histogram), these tools typically lack a visual output, such as plots, and/or do not offer an all-in-one solution to accomplish these tasks in a single run.

Currently, to evaluate common length distributions and retrieve sequences for analyses like *de novo* transcriptome assembly, ORF translation, or bioactive peptide discovery using thresholds such as 200 bp, 100 aa, or 40–60 amino acids, researchers often rely on a combination of shell or Python commands with tools like SeqKit.

Considering that many of these tasks are handled by separate tools, we introduce **SeqLengthPlot v2.0**: a comprehensive and easy-to-use Python-based tool that combines these functionalities into a single unified tool. In this version, we have implemented command-line flags for improved flexibility, allowing users to easily customize their analysis parameters, visualize sequence length distributions, and retrieve sequences based on specified cutoffs. Herein, we present the implementation, features, outputs, and applications of **SeqLengthPlot v2.0**, tested on original data from the single-end transcriptome of the false black coral *Savalia savaglia* (Cnidaria) across the most common operating systems.

## 2 Tool description

**Figure 1.**
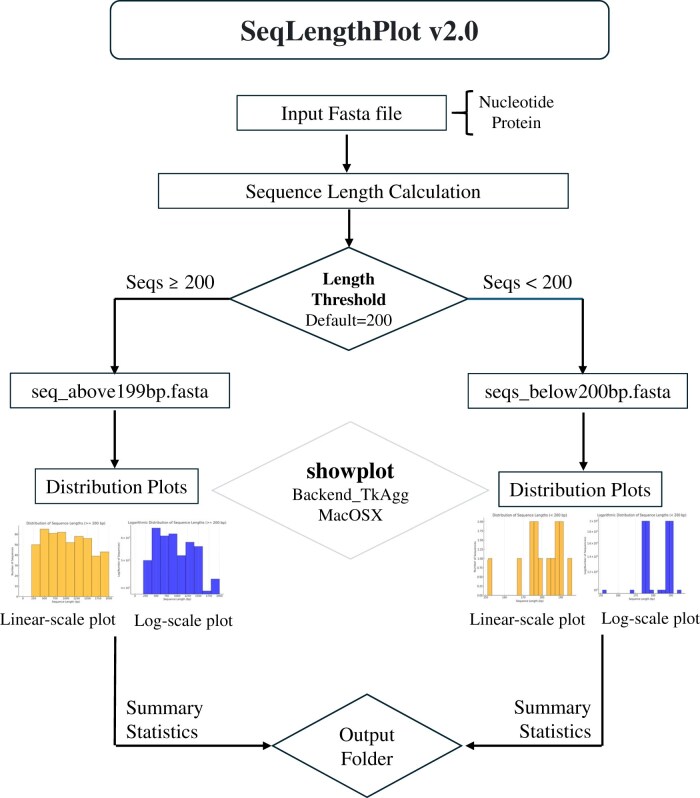
Illustrative diagram of the workflow and main components of SeqLengthPlot v2.0. The workflow begins with inputting a FASTA file containing nucleotide or protein sequences. The user can specify parameters such as input files, output directories, cutoff lengths, and sequence types through command-line flags. SeqLengthPlot then processes the sequences by splitting them into two categories: sequences above and below the specified cutoff length. Finally, the tool generates detailed outputs, including statistical summaries and visual plots of sequence length distributions in both linear and logarithmic scales.

The **SeqLengthPlot** is an all-in-one, easy-to-use Python-based tool for visualizing and retrieving sequence lengths from FASTA files. It splits sequences by length threshold, generates distribution plots, and provides detailed statistics (Fig. 1). The new **SeqLengthPlot v2.0** allows users to fully take advantage of its functionalities through simple and customizable command-line flags.

### 2.1 Key features

- **Comprehensive metrics and output**: Unlike other tools, SeqLengthPlot offers a complete solution in a single tool, providing the total number of sequences, as well as the minimum and maximum lengths of both the input and split files. In addition, it generates the corresponding FASTA files, containing sequences that are below and above the specified cutoff.
- **Visual analysis**: SeqLengthPlot generates intuitive plots for sequence length distributions, offering both **linear** and **logarithmic** scales. It supports both interactive explorations and automated pipelines, accommodating a wide range of sequence lengths and enhancing data interpretation for various analyses.
- **Flexible threshold settings**: The tool allows users to set custom length cutoffs, which is critical for tasks like validating *de novo* transcriptome assemblies, ORFs translation, and exploring peptidomics or bioactive peptide discovery, which may require specific length criteria.
- **New command-line flexibility**: With **version 2.0**, the tool now includes customizable **command-line flags**, allowing users to easily adjust input files, output directories, sequence types (nucleotide or protein), cutoff lengths, and backend plotting options, enhancing both usability and flexibility.
- **Ease of integration**: SeqLengthPlot can be run as a standalone script or incorporated into larger bioinformatics workflows. It supports both interactive explorations and automated pipelines, making it suitable for a wide range of bioinformatics applications.

### 2.2 Applications

- **Transcriptome integrity and sequence length distribution**: SeqLengthPlot assists in evaluating the accuracy of common cutoff lengths (e.g. 200 bp) used by RNAseq assemblers such as **Trinity**. Researchers can assess the distribution and abundance of transcripts shorter or longer than 200 bp (Fingerhut *et al.* 2018, Almeida *et al.* 2020), as well as higher frequency peaks in the length histogram (e.g. near the N50 value), which helps evaluate the overall assembly quality. This assessment also aids in deciding whether to remove shorter sequences before or after assembly, or for database submissions, such as the **Transcriptome Shotgun Assembly (TSA)** database.
- **Exploring ORF and peptide lengths:** Analyze the occurrence and distribution of open reading frames (ORFs) and peptides that fall below or exceed 100 amino acids, a common threshold used by **TransDecoder** (Haas and Papanicolaou 2024) users for protein annotation (Fingerhut *et al.* 2018, Almeida *et al.* 2020).
- **Characterizing peptide lengths for bioactive discovery**: Investigate the distribution, splitting, and automatic retrieval of peptides generated by tools like **TransDecoder** (Haas and Papanicolaou 2024), **six-frame translation tools** (Rice *et al.* 2000), **orfipy** (Singh and Wurtele 2021), and DeTox (Ringeval *et al.* 2024). This assessment enhances the accuracy and classification of bioactive peptide classes from the split files, as certain peptides or machine learning algorithms are optimized for specific length ranges (Agüero-Chapin *et al.* 2024).

### 2.3 Compatibility, installation, and dependencies

**SeqLengthPlot v2.0** is compatible with both Unix and Windows operating systems. To run it, ensure you have an updated version of Python installed and dependencies for plotting and sequence manipulation.

**Dependencies:**

- **Python 3.x**
- **Matplotlib**
- **Biopython**

Refer to our comprehensive guide on GitHub for detailed instructions on installing Python and dependencies across various configurations.

#### 2.3.1 Command-line flags in SeqLengthPlot v2.0

**Mandatory flags:**

**-i (input file)**: The path to the input FASTA file (this is required).

**Optional flags:**

**-o (output directory)**: If not provided, a folder is automatically generated in the input file’s directory based on the input FASTA file's name, sequence type, and threshold.
**--cutoff (cutoff):** The default sequence length cutoff is 200. You can adjust this with the --cutoff flag.
**--nt:** Specifies nucleotide sequences (default behavior).
**--prot:** Specifies protein sequences (changes file extensions to .aa).
**--showplot:** If this flag is used, plots will be displayed interactively. Otherwise, they are saved but not shown.
**--backend:** Specify the plotting backend for matplotlib (TkAgg for Linux/Windows or MacOSX for macOS). The default is TkAgg.

### 2.4 How to run SeqLengthPlot_v2.0

- Go to the GitHub SeqLengthPlot repository: https://github.com/danydguezperez/SeqLengthPlot, and download the latest release. After downloading it, SeqLengthPlot_v2.0.2.zip, unzip the file.
- Navigate to the directory where SeqLengthPlot_v2.0.2.py is located:
  cd/path/to/SeqLengthPlot_v2.0.2.py
- Run the script with the required input file:
  python SeqLengthPlot_v2.0.2.py -i input.fasta

The “**-i**” flag and the corresponding input.fasta is the only mandatory parameter.

**If the FASTA file is not provided, the script will return the following error message:**

usage: SeqLengthPlot_v2.0.2.py [-h] -i INPUT [-o OUTPUT] [--cutoff CUTOFF] [--nt] [--prot] [--showplot] [--backend BACKEND]

SeqLengthPlot_v2.0.2.py: error: the following arguments are required: -i/--input

**If the flag “-i” is provided but the path to the input.fasta file given is incorrect, the script will return the following error message:**

An error occurred: [Errno 2] No such file or directory.

**For Help or Command-Line Flags Information:**

To get more information on available flags and options, use the **-h** flag:

- python SeqLengthPlot_v2.0.2.py -h

## 3 Examples of a real-usage cases

### 3.1 Testing SeqLengthPlot v2.0.2 on transcriptomic data

**Figure 2.**
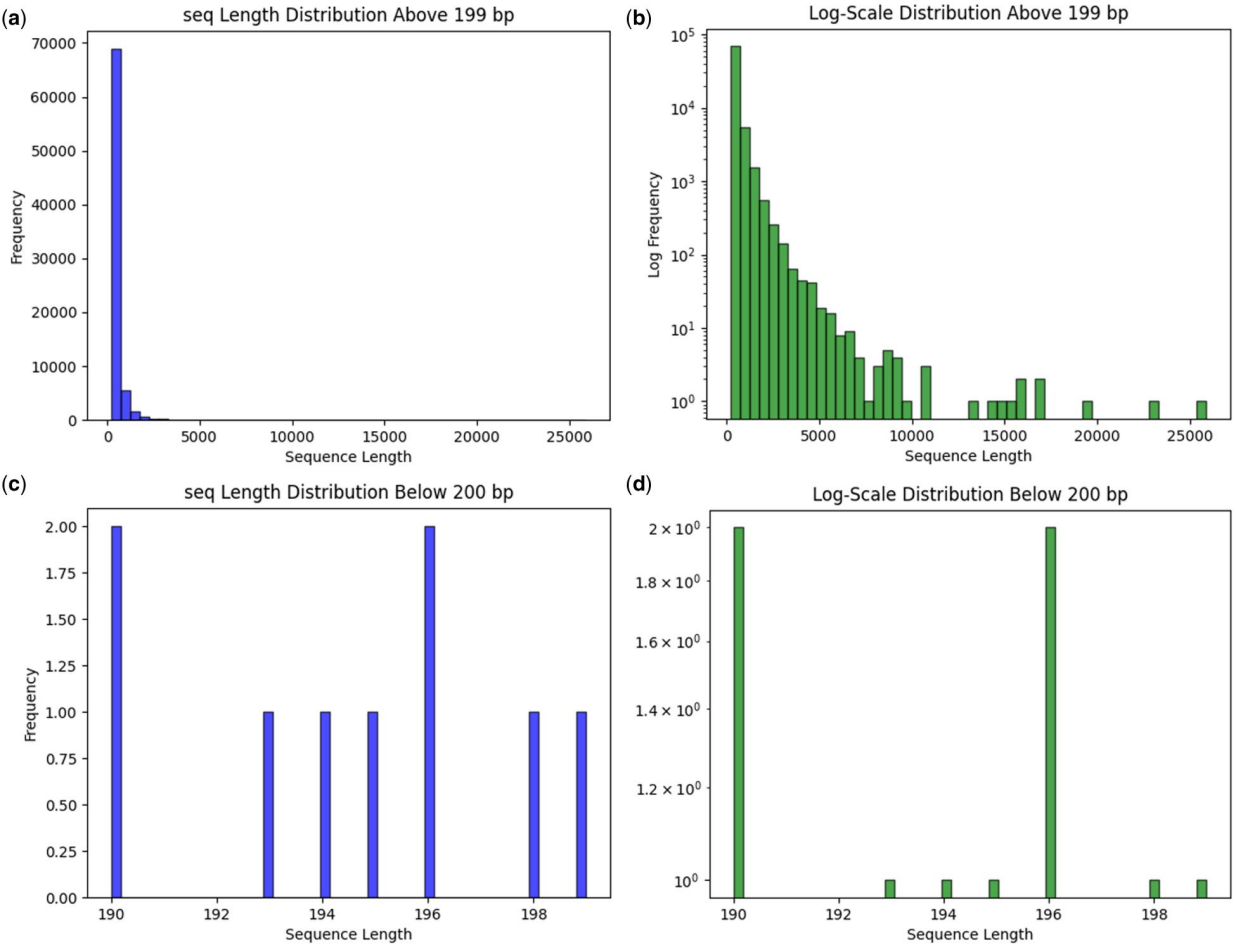
Sequence length plot of the single-end transcriptome of *Savalia savaglia* (Cnidaria) based on a 200 bp cutoff. Panels (a) and (b) display the length distribution of sequences above 199 bp in standard and log scales, respectively. Panels (c) and (d) show the length distribution of sequences below 200 bp in standard and log scales.

Here, we test SeqLengthPlot v2.0.2 on original data obtained from the single-end transcriptome of the false black coral *S. savaglia* (Cnidaria), using providing only the mandatory parameter. The input FASTA file, plots (Fig. 2) and all the files generated can be found within a compressed folder Example_SeqLengthPlot_v2.0.2.zip, latest releases (Releases v2.0.2) here https://github.com/danydguezperez/SeqLengthPlot.

python SeqLengthPlot_v2.0.2.py -i **Assembly_Ss_SE.Trinity.fasta**

**Input file:**

- **Input.fasta** file: nucleotide (Assembly_Ss_SE.Trinity.fasta)

**Outputs files (using sequence length = 200bp, default cutoff):**

- **Fasta files**: Two FASTA files (**seq_above199bp.fasta** and **seqs_below200bp.fasta**), splitted and retrieved from the orginal **input_fasta** file categorizing sequences based on the length threshold.
- **Histogram plots**: Four PNG files showing histograms of sequence lengths. Two are in standard scale (**seq_length_distribution_above199bp.png** and **seq_length_distribution_below200bp.png**), and two are in log scale (**seq_length_distribution_above199bp_log.png** and **seqs_length_distribution_below200bp_log.png**).
- **Statistical summary**: A text file (**seq_length_stats_by_threshold_200.txt**) containing detailed statistics of the sequence lengths on the **input.fasta**: Total number of input Sequences, Number of Sequences above 199 bp and below 200 bp, with the corresponding minimum and maximum lengths.

### 3.2 Example of a full command line for protein sequences

Here, we test SeqLengthPlot v2.0 on toxin candidates identified by DeTox in the single-end transcriptome of *S. savaglia*

**python** SeqLengthPlot_v2.0.2.py **-i** Assembly_Ss_SE.Trinity.fasta **-o**/path/to/output_directory --**cutoff** 100 --prot --showplot --backend MacOSX

**Explanation:**

**-i input.fasta**: Specifies the input FASTA file (mandatory parameter).
**-o/path/to/output_directory**: Specifies the directory where output files (FASTA, plots, stats) will be saved. If omitted, the files will be saved in the input file's directory.
**--cutoff 100**: Sets the cutoff for sequence length. Sequences will be split based on a length of **100 aa** (amino acids), instead of **200 bp** (base pairs) for nucleotide sequences (default cutoff), since --**prot** is specified in the command line for protein sequences in the input FASTA file.
**--prot**: Specifies that the input file contains **protein sequences**. When this flag is used, output files, plots, legends, and stats will be generated with **.aa** (amino acids) as the unit. If omitted, the default is nucleotide sequences (**--nt**), and the output will use **.bp** (base pairs).
- **“--showplot”**: Displays the plots interactively. If this flag is not included, the plots will be saved but not shown.
**--backend MacOSX**: Specifies the backend for plotting. Use “**MacOSX”** for macOS users, instead of default “**TkAgg,”** for Linux/Windows.

## Data Availability

The code and data underlying this article are available on GitHub in the SeqLengthPlot repository: https://github.com/danydguezperez/SeqLengthPlot. The latest version presented here (SeqLengthPlot_v2.0.2) can be accessed at https://github.com/danydguezperez/SeqLengthPlot/releases/tag/v2.0.2. The example data used in this article can be found at https://github.com/user-attachments/files/17356731/Example_SeqLengthPlot_v2.0.2.zip.

## References

[vbae183-B1] Agüero-Chapin G , Domínguez-Pérez D, Marrero-Ponce Y et al Unveiling encrypted antimicrobial peptides from Cephalopods’ salivary glands: a proteolysis-driven virtual approach. ACS Omega 2024;9:43353–67. 39494035 10.1021/acsomega.4c01959 PMC11525497

[vbae183-B2] Almeida D , Domínguez-Pérez D, Matos A et al Data employed in the construction of a composite protein database for proteogenomic analyses of cephalopods salivary apparatus. Data 2020;5:110.

[vbae183-B3] Fingerhut LCHW , Strugnell JM, Faou P et al Shotgun proteomics analysis of saliva and salivary gland tissue from the common octopus *Octopus vulgaris*. J Proteome Res 2018;17:3866–76. 30220204 10.1021/acs.jproteome.8b00525

[vbae183-B4] Grabherr MG , Haas BJ, Yassour M et al Full-length transcriptome assembly from RNA-Seq data without a reference genome. Nat Biotechnol 2011;29:644–52. 21572440 10.1038/nbt.1883 PMC3571712

[vbae183-B5] Haas BJ , Papanicolaou A, Yassour M et al De novo transcript sequence reconstruction from RNA-seq using the Trinity platform for reference generation and analysis. Nat Protoc 2013;8:1494–512. 23845962 10.1038/nprot.2013.084 PMC3875132

[vbae183-B6] Haas BJ , Papanicolaou A. TransDecoder 5.7. 1. https://github.com/TransDecoder (22 May 2024, date last accessed).

[vbae183-B7] Rice P , Longden I, Bleasby A et al EMBOSS: the European molecular biology open software suite. Six-frame translation tool. Trends Genet 2000;16:276–7. 10827456 10.1016/s0168-9525(00)02024-2

[vbae183-B8] Ringeval A , Farhat S, Fedosov A et al DeTox: a pipeline for the detection of toxins in venomous organisms. Brief Bioinform 2024;25:bbae094. 38493344 10.1093/bib/bbae094 PMC10944572

[vbae183-B9] Shen W , Le S, Li Y et al SeqKit: a cross-platform and ultrafast toolkit for FASTA/Q file manipulation. PLoS One 2016;11:e0163962. 27706213 10.1371/journal.pone.0163962 PMC5051824

[vbae183-B10] Singh U , Wurtele ES. orfipy: a fast and flexible tool for extracting ORFs. Bioinformatics 2021;37:3019–20. 33576786 10.1093/bioinformatics/btab090 PMC8479652

[vbae183-B11] Telatin A , Fariselli P, Birolo G et al SeqFu: a suite of utilities for the robust and reproducible manipulation of sequence files. Bioengineering 2021;8:59. 34066939 10.3390/bioengineering8050059 PMC8148589

